# A Safe Fiber-Optic-Sensor-Assisted Industrial Microwave-Heating System

**DOI:** 10.3390/s24216995

**Published:** 2024-10-30

**Authors:** Kivilcim Yüksel, Oguz Deniz Merdin, Damien Kinet, Murat Merdin, Corentin Guyot, Christophe Caucheteur

**Affiliations:** 1 Electronics Engineering Department, Izmir Institute of Technology, Urla, TR-35430 Izmir, Türkiye; 2 MET Advanced Technologies, TR-35430 Izmir, Türkiyemurat.merdin@metltd.net (M.M.); 3 B-SENS, Boulevard Dolez 31, 7000 Mons, Belgium; damien.kinet@umons.ac.be (D.K.); corentin.guyot@b-sens.be (C.G.); 4 Advanced Photonic Sensors Unit, University of Mons, Boulevard Dolez 31, 7000 Mons, Belgium; christophe.caucheteur@umons.ac.be

**Keywords:** microwave oven, heating, drying, fire detector, fiber-optic sensors, fiber Bragg gratings (FBGs)

## Abstract

Industrial microwave-heating systems are pivotal in various sectors, including food processing and materials manufacturing, where precise temperature control and safety are critical. Conventional systems often struggle with uneven heat distribution and high fire risks due to the intrinsic properties of microwave heating. In this work, a fiber-optic-sensor-assisted monitoring system is presented to tackle the pressing challenges associated with uneven heating and fire hazards in industrial microwave systems. The core innovation lies in the development of a sophisticated fiber-optic 2D temperature distribution sensor and a dedicated fire detector, both designed to significantly mitigate risks and optimize the heating process. Experimental results set the stage for future innovations that could transform the landscape of industrial heating technologies toward better process quality.

## 1. Introduction

Microwave heating is renowned for its efficiency (lower energy consumption) and speed (shorter heating times), contrasting with conventional heating methods. Microwave-heating technology is enhancing energy efficiency and product quality in food processing [1] and materials science [2]. It also provides the ability to selectively heat different components within a product streamline and reduces thermal gradients [3].

Despite its advantages, the uneven distribution of microwave energy remains a critical challenge. The non-uniform heating can lead to hotspots that degrade product quality and increase the risk of thermal runaway reactions, potentially leading to fires in industrial settings [2]. These challenges underscore the need for advanced monitoring and control systems within microwave ovens [4,5]. To monitor the temperature distribution of ovens, infrared or thermal camera monitoring may be used. However, they cannot operate effectively inside high-electromagnetic-field environments. Hence, sensing microwave energy using fiber-optic probes has become an option [6]. For fire detection, smoke sensors or relative humidity sensors are usually used inside microwave ovens. High water vapor from products inside ovens generally causes malfunctions on the sensor. There are no specific fire detection systems for industrial microwave ovens [7,8]. For all these reasons, it is highly relevant to develop fiber-optic sensors for industrial microwave ovens.

Among all fiber optic sensors, fiber Bragg gratings (FBGs) have great advantages, such as their wavelength-encoded linear response (not influenced by power fluctuations), multiplexing capabilities (hence, interrogation of tens of FBGs by a single interrogation unit), easy implementation (i.e., can be attached or embedded to the material), and cost effectiveness (thanks to mass production and multiplexing) [9,10,11]. They are also suitable to be used in harsh environments [12].

FBGs have been used as temperature sensors in specially designed glass pipes to measure the liquid temperature in a household microwave oven [13]. In another study, FBG sensors were implemented in a coiled flow channel inside a microwave reactor to monitor the temperature distribution [14]. In the latter example, FBG sensors were interrogated using a rather expensive optical frequency domain reflectometer (OFDR). Moreover, none of those works was focused on industrial microwave ovens. They are rather laboratory-type prototypes implemented on small commercial microwave devices modified for the experiments. Seamless implementation of these FBG sensors in the industrial microwave ovens, however, brings about many design challenges. Sensors should be properly designed (fiber type, gauge-length, etc.) and protected (coated). Also, the number of sensors (i.e., the distance between the sensors) that should be distributed over the oven cavity should be optimized. To the best of our knowledge, there has been so far, no published design recipe and/or commercial product to answer these design issues for the very specific application of microwave-heating systems.

The primary objective of this work is to address abovementioned issues by implementing fiber-optic sensors capable of real-time temperature monitoring and the early detection of fire hazards within industrial microwave systems. In our highly applied experiments, FBG-based temperature sensors and fire detectors have been developed and integrated in a smart monitoring platform. The feasibility of the proposed platform has been successfully demonstrated.

## 2. Instrumentation and Calibration

**Figure 1:**
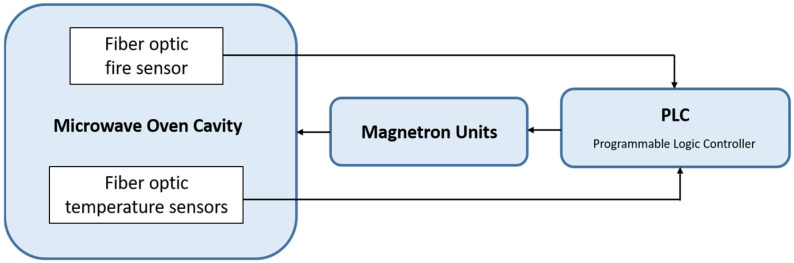
Block diagram of the sensor-assisted microwave-heating system.

Figure 1 depicts a block diagram of the microwave-heating system consisting of a microwave cavity with its microwave generation and control units, an optical interrogator, fire detector, and optical fibers with the FBG temperature sensors.

Fiber-optic 2D temperature distribution sensors designed and fabricated by B-Sens (www.b-sens.be) (accesses on 5 September 2024) have been mounted inside the microwave cavity. Using this temperature data, a closed loop PID (Proportional–Integral–Derivative) feedback system has been designed, where programmable logic controllers process these data. With this closed loop system, energy consumption and process time significantly decreased.

MET company (https://metltd.net/en) (accesses on 5 September 2024) was responsible for 3D microwave cavity design, prototype cavity production, fire starter setup, fire extinguisher setup, and electromagnetic simulations and apparatus for sensor mounting and positioning.

For a given measurement, a 16-channel interrogator measures and processes the outputs of all the FBG temperature sensors and the fire detector simultaneously. This interrogator, comprising a broadband optical source and a 512-pixel spectrometer, can measure up to 40 sensors on a single channel (the wavelength range is between 1510 and 1590 nm). Its frequency sweep can be modified from 1 Hz up to 3 kHz over one channel.

### 2.1. Fabrication of Fiber Bragg Gratings

Two different FBG inscription methods were used in this work. A Lloyd setup was used for the fabrication of temperature sensors, while a phase-mask technique was preferred for IR detectors. This decision was taken mainly due to the wavelength-tuning capability of the former (i.e., Lloyd setup), which is required for the temperature sensor grid (tens of different FBGs on the same fiber with different Bragg wavelengths).

1—Phase mask technique: The inscription by the phase mask is performed using a continuous laser whose wavelength is 244 nm (UV). A phase mask is a pattern of grooves etched into a UV-transmitting pure silica plate that diffracts the incident laser beam mainly in the + and −1 orders, inducing interference at the core of the optical fiber (previously located at the right distance). The Bragg wavelength is directly linked to the phase mask period. Hence, this technique allows for rapid inscription with good reproducibility of Bragg gratings, but it is not the best-suited to produce Bragg gratings at any wavelength.

2—Lloyd’s interferometer: The second method consists of photo-inscribing the Bragg grating at the core of the fiber using the so-called Lloyd’s mirror technique. Similar to phase mask technique, a 244 nm continuous UV laser is used. The main difference is that, instead of using a phase mask, the interference at the fiber core is created by dividing the laser beam into two paths (having a slightly different optical path induced by the reflection of the beam on a mirror). The optical path difference can be modified by modifying the orientation of the mirror. This in turn changes Bragg gratings’ optical response in reflection. With this method, it is possible to precisely choose the reflection wavelength of the Bragg grating we want to create.

For both techniques, germanium-doped optical fibers are used. Indeed, these defects (doping), present in the core of the fiber, will make it possible to locally modify the refractive index under the illumination of the UV laser during the photo-inscription (photosensitivity). These two methods provide uniform Bragg gratings whose reflectivity can be adjusted by illuminating the fiber core with a UV laser for a longer or shorter time (duration of the exposure).

Packaging of FBG temperature sensors: Various materials were tested for packaging the FBGs, including PTFE, ETFE, and Hytrel, to ensure they did not disturb the electromagnetic distribution inside the oven and were inert to microwaves. The selected material, PTFE, demonstrated optimal performance in thermal stress tests and was chosen for subsequent phases.

Calibration efforts focused on distinguishing between temperature and strain effects on the Bragg wavelength shift, as both can influence the FBG response. It was essential to ensure that the FBGs were free of axial strain to accurately measure temperature within the microwave oven.

### 2.2. Calibration of FBG Sensors

**Figure 2:**
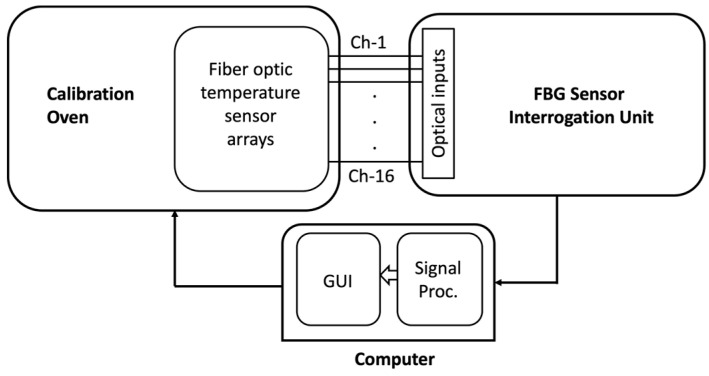
Schematic representation of sensor calibration setup.

The experimental set-up that was used to calibrate the sensors (between room temperature up to 80 °C) and determine the temperature sensitivity is shown in Figure 2 [15]. A Memmert UF55plus appliance (Memmert, Germany) was used for calibration, equipped with a stainless-steel door and two reference thermocouples to provide a controlled and homogeneous heating environment. The appliance was modified with a 38 mm entry port to accommodate the fibers without compromising the heat distribution or damaging the delicate sensors. After having the FBG arrays (each fiber containing 16 FBGs) placed in the calibration oven (Memmert UF55plus), the fiber ends were connected to the 16-channel FBG sensor interrogator unit. A computer was used to command the oven with predefined temperature heating cycles, process the measurement results, and display the calibration curves.

**Figure 3:**
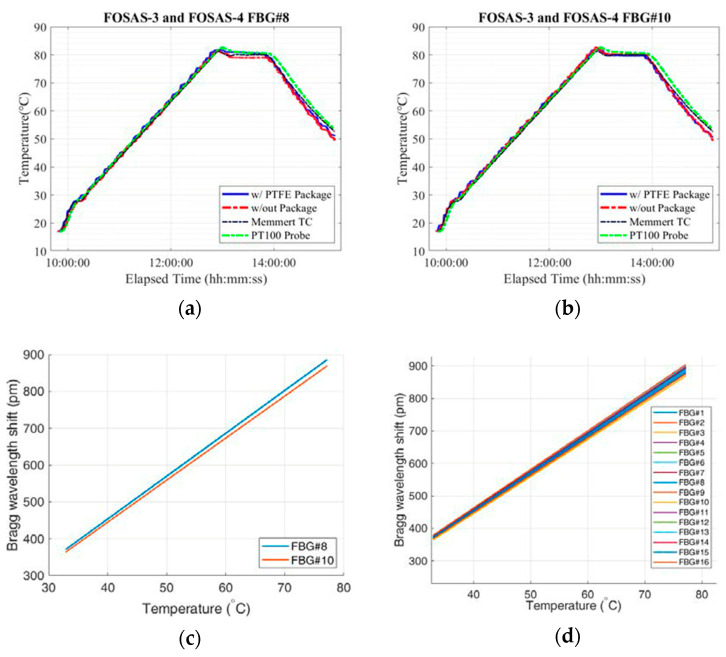
Variation in temperature measured using reference probes and FBG sensors (both with and without package) during a temperature cycle (from room temperature to 80 °C) for FBG#8 (**a**) and FBG#10 (**b**). Variation in the Bragg peak position as a function of the temperature for FOSAS-4 (having PTFE package), FBG#8, FBG#10 (**c**), and FOSAS-4 all 16 FBGs (**d**).

The results for two of the FBG arrays (i.e., FOSAS-3 and FOSAS-4) can be found in the graphs presented in Figure 3 (among 16 FBGs characterized individually, only 2 FBGs, namely FBG#8 and FBG#10, are represented here. Other FBGs in the fibers react in the same way). In Figure 3a,b, the green (Pt100) and black (Memmert TC) lines show the variation in temperature measured with two thermocouple probes over a temperature cycle between room temperature and 80 °C. These probes will act as a reference with respect to the Bragg grating sensors. We note on the same graphs that the temperature value measured by the Bragg grating sensors follow the variation in the temperature measured using the probes. Also, we observe that the response of the sensors without package (red line) fluctuates more than that of packaged sensors (blue line). Using these measurements, linear response of the FBGs (calibration curves) are obtained for each sensor individually, as represented in Figure 3c,d.

Sensitivity: There are two factors leading to the temperature sensitivity of an FBG: the grating period variation (thermal expansion coefficient of the optical fiber) and the refractive index change (thermal dependence of the refractive index). The temperature sensitivity of the FBG can be expressed as the amount of Bragg wavelength shift per change in temperature (i.e., slopes of linear lines in Figure 3c,d).

Measurements necessary to determine the sensitivity values of each FBG have been repeated several times. Then, the mean and variance of the sensitivity values have been calculated. Some tests were conducted on the same day (sequential heating cycles), while the others were organized on different days to take variations in the laboratory conditions into account. Sensor arrays have been heated and left to return to room temperature over sequential cycles. The average sensitivity values measured during repeatability tests vary between 11 pm/°C and 11.7 pm/°C, which is consistent with values previously reported for highly doped Ge fiber. The maximum standard deviations of the sensitivities belonging to the unpackaged and PTFE-packaged sensor arrays are 0.42 pm/°C and 0.17 pm/°C, respectively. Packaging has been shown to enhance the stability of the sensor response as expected.

### 2.3. Design of the Fire Detector

The effectiveness of FBG sensors in detecting infrared (IR) radiation emitted by hot spots is critical for the early detection of fires. IR emissions from typical hot spots range from 0.8 μm to 15 μm, with the most significant emissions occurring below 4 μm for temperatures around 1500 K, characteristic of flaming fires. Given that silica (SiO_2_), the primary material of the FBG, does not absorb IR radiation in this critical range, enhancing the FBG’s sensitivity through functional coatings was essential.

The early design examples of fiber bolometers for the purpose of IR radiation detection were demonstrated in [16,17,18]. Inspired by the traditional bolometer design, a fiber bolometer implements a pair of FBGs; one of the FBGs is coated with a special material to absorb IR radiation, while the other remains uncoated to serve as a temperature reference. IR radiation is converted into heat by the coated FBG. Therefore, when subject to IR radiation, the temperature of the FBG under exposure will increase, and its Bragg wavelength will be shifted. IR detection sensor using fibered bolometers as sensing points can be investigated by using a power-meter (the total reflected power from the FBG pair is measured), by using an optical spectrum analyzer (OSA), or an OFDR (optical frequency domain reflectometer) [18].

In our monitoring system, we implemented a fiber bolometer to design a fire detector. One of the FBGs in the FBG pair was coated with CuO to absorb IR radiation (the other FBG was used as temperature reference). This setup facilitated the comparison of temperature-induced wavelength shifts between the exposed and reference FBGs, enhancing the accuracy of fire detection.

Coating materials and processes:

- Copper (II) Oxide (CuO) coating: Chosen for its strong IR absorption below 5 μm, CuO coatings were applied to the FBGs to increase their sensitivity to thermal radiation. This coating effectively converts IR radiation into detectable thermal changes, evidenced by shifts in the FBG’s Bragg wavelength.
- Application method: The CuO was dispersed in a polymer matrix and applied to the FBGs using a dip-coating technique, which allowed for the formation of a uniform, thin layer essential for precise sensor responses. This layer is very stable over time. It sticks firmly to the optical glass fiber and is mechanically stable.

**Figure 4:**
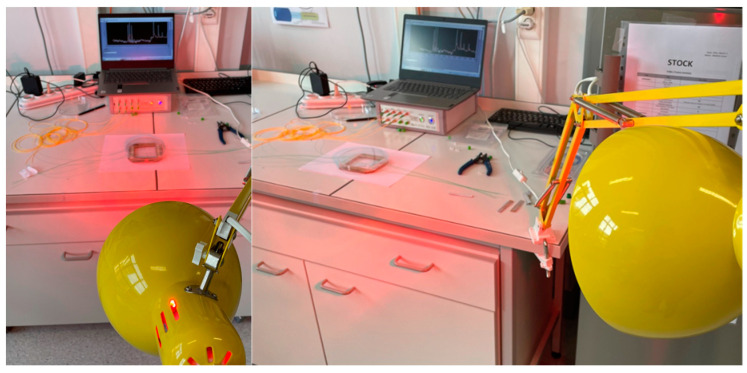
Photo showing the setup for the functional tests of the IR detector. (**left**): direct exposure, (**right**): angled exposure. The distance between the sensor and the IR source is varied between 15 cm and 1 m.

**Figure 5:**
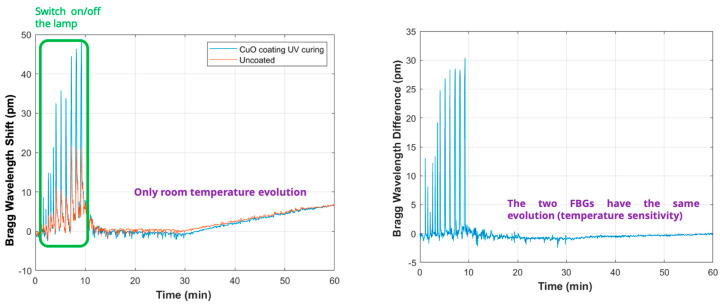
IR detector response: (**left**): Bragg wavelength shift (BWS), (**right**): Bragg wavelength difference (BWD). Time of exposure is varied between 1 s and 10 s.

Prior to implementation into the microwave oven, the configurations (the distance between the coated and the reference FBGs, IR exposure time, IR exposure distance) were tested in the laboratory environment (cf. Figure 4). The FBGs were configured in pairs spaced by 30 mm and 50 mm to test different response times and sensitivity levels. The experimental setup included an IR source (Philips IR250R lamp) positioned at varying distances to adjust the radiation flux experienced by the sensors. Then, the effectiveness of the coatings was assessed by monitoring the shift in the Bragg wavelengths of the FBGs using a customized FBG interrogator. This method allowed for precise tracking of the changes in reflected wavelengths as the FBGs responded to IR exposure. Among a large variety of trials, let us present an example test case, where the lamp is switched ON and OFF during the first 10 s of the measurement. The IR detector response is presented in Figure 5.

**Figure 6:**
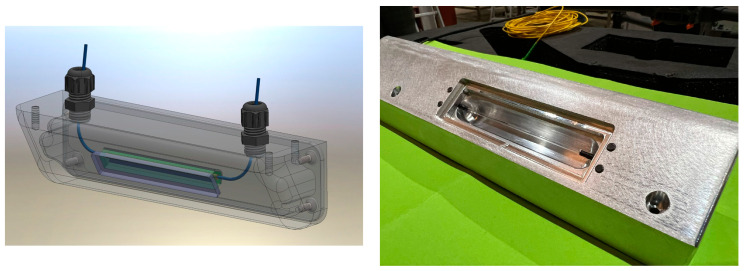
Manufactured casing for fire sensor.

After preliminary laboratory tests, a specialized casing was developed and integrated within the microwave oven cavity (cf. Figure 6). This casing was designed to protect the sensor and enhance its effectiveness in detecting fires. In the design and implementation of fire detection systems within industrial settings, the positioning of the sensor casing is crucial for ensuring comprehensive coverage and maximum sensitivity. In this project, the casing for the fire sensor was deliberately placed at the middle top of the cavity. This strategic placement was chosen to capitalize on several key factors that contribute to the effective monitoring of fire conditions throughout the oven cavity.

### 2.4. Design and Production of Microwave Oven Modules

In the framework of this work, a new cavity design was realized for microwave generation. The new microwave oven cavity design significantly improved upon the old design, evidenced by the S-parameter measurements which showed enhanced performance. The term “S-parameter”, or scattering parameter, is commonly used in the context of microwave engineering to describe how radio frequency (RF) signals behave when they encounter different electrical networks. It quantifies how much of an incident signal is transmitted, reflected, or absorbed by a device, providing critical information about system performance, especially in terms of signal integrity.

The new design achieved S-parameters below −15 dB for all magnetrons, with eight of them performing around −20 dB at 2.45 GHz. This indicates a more efficient transmission of energy and less signal loss.

The updated cavity design not only accommodated a higher power acceptance, increasing from 15,242.53 W to 15,484.6 W, but also improved the power transmission to water from 88.04% to 92.02%. Despite a slight increase in the loss in dielectric material, from 14,087.97 W to 14,724.24 W, the overall efficiency and effectiveness of the microwave heating were significantly enhanced.

**Figure 7:**
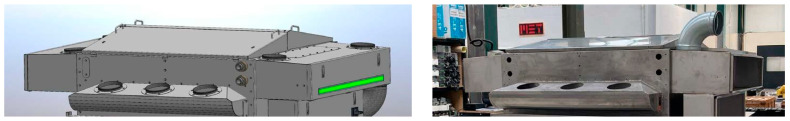
Mechanical design and the manufactured cavity.

Figure 7 illustrates the mechanical design and the actual manufactured new cavity, respectively. These images highlight the tangible outcomes of the design improvements and serve as a visual confirmation of the enhancements. Based on the detailed electrical scheme, a suitable cabinet trunk was chosen for housing the new, energy-efficient microwave drivers. The selection criteria focused on capacity, safety, and efficiency to accommodate the specific needs of 16 magnetron drives, which are crucial for generating the microwave energy.

A newer type of microwave driver was incorporated to boost the system’s overall energy efficiency. These drivers are essential for reducing power consumption while maintaining high operational standards. To optimize cabling and minimize signal loss and electromagnetic interference, the microwave drivers were strategically positioned on either side of the microwave cavity. This layout not only improved the efficiency of cable routing but also enhanced the system’s overall electromagnetic compatibility.

### 2.5. Data Acquision and Analysis

The project partners collaborated to develop a new data acquisition program tailored to B-Sens’ interrogators, which provided extended accessibility. The newly developed software enables starting the test cycle through a PC interface, which simultaneously initiates data acquisition from the interrogators and activates the test cycle on the PLC. This integration allows for precise control over the testing process, including the ability to stop the cycle as required. Consequently, these halt both the microwave activity and data acquisition, ensuring safety and efficiency.

## 3. Measurement Results

**Figure 8:**
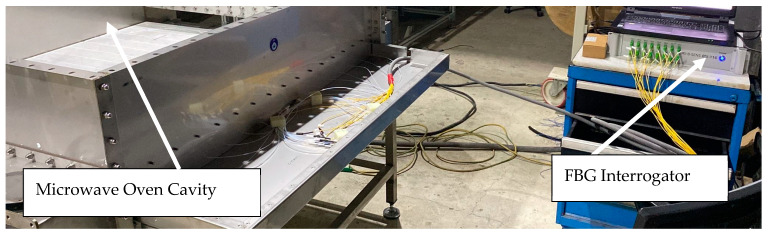
Measurement setup with the instrumented microwave oven on the left and the BSI-116 data acquisition system on the right.

The modified microwave system underwent extensive testing to evaluate the performance of the integrated sensors (cf. Figure 8). Tests included controlled-heating trials to assess the uniformity of temperature distribution and response times of the fire detection system.

Additional tests were conducted to verify the system’s safety and efficacy in real-world industrial conditions. These trials aimed to confirm that the integrated sensors could effectively prevent overheating and fire hazards during normal operations.

### 3.1. Selection of Test Material

The process of selecting an appropriate medium for placing the FBG sensors in the microwave oven involved several experimental trials with various materials (i.e., agar agar, water, salami, marble, etc.) Marble, as a durable and stable material, was reconsidered, given its known properties of good heat retention and its capability to be precisely machined. Before finalizing marble as the medium, we conducted preliminary tests with a new cavity design to evaluate its interaction with microwave energy. We found that marble is an excellent candidate for securely placing fiber optics in the desired pitch due to its machinability, ensuring precise dimensions. Additionally, marble’s lack of water content eliminates any risk of hindering the fiber optics. It exhibits very minimal dimensional changes when subjected to heat and has good heating characteristics under microwave conditions, validating its use for our application.

**Figure 9:**
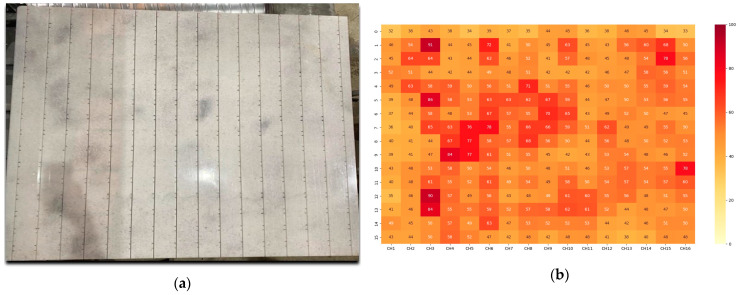
(**a**) Position of veins; (**b**) marble heating (a temperature difference up to 57 °C was observed between the hot spots of marble’s natural veins and the marble’s white parts). The cavity was 1500 mm × 1500 mm.

The marble surface and its heating pattern are represented in Figure 9a,b, respectively. The marble surface is equipped with 16 fibers (vertical lines represented in Figure 9a), each containing 16 FBGs. The absolute temperature values measured by this 16 × 16 temperature sensor grid are represented in Figure 9b. The first set of tests with the new cavity highlighted a non-homogeneous heating pattern. The marble’s natural veins heated differently compared to its whiter parts, providing critical insights into the material’s interaction with microwave energy. As can be seen from Figure 9b, vein regions heated up to around 90 °C, while the temperature values in the rest of the marble block varied between 33 and 50 °C.

To further investigate these heating discrepancies, the marble slab was rotated within the cavity. This adjustment aimed to determine if the heating would become more uniform when the marble was oriented differently. The results demonstrated that the new cavity could consistently heat the marble evenly in both original and rotated 180-degree orientations, affirming the effectiveness of the chosen setup.

### 3.2. Comparison of Temperature Profiles of Old and New Cavities

The old and new cavities were tested under similar conditions, and the results showed lower temperature values along the center lines of the new cavity, aligning with the predictions from the simulations. These tests further validated the improvements in the new cavity design. Additional tests were conducted for durations of 30, 60, 90, and 360 s, with the marble cooled down to 15–16 degrees Celsius in between tests. Subtraction of the temperatures is made with the differences in start temperature and end temperature of the marble for 30, 60, 90, and 360 s.

**Figure 10:**
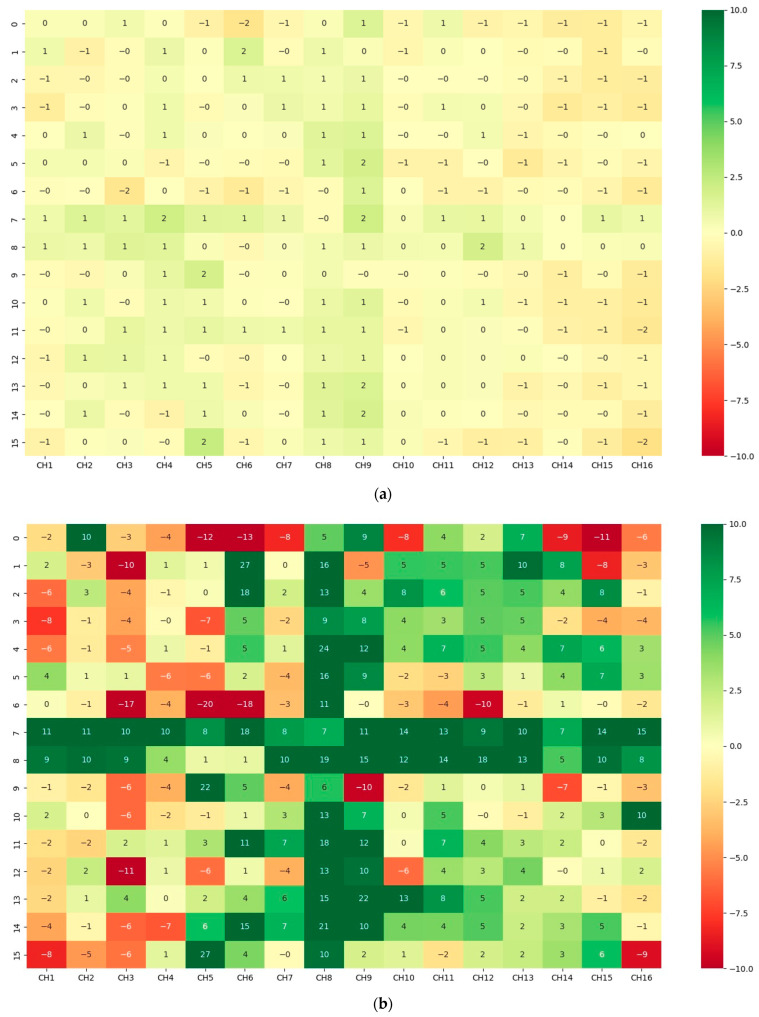
(New cavity/old cavity) 2D heating-difference results (**a**) at 30 s and (**b**) at 360 s. The cavity is 1500 mm × 1500 mm.

**Figure 11:**
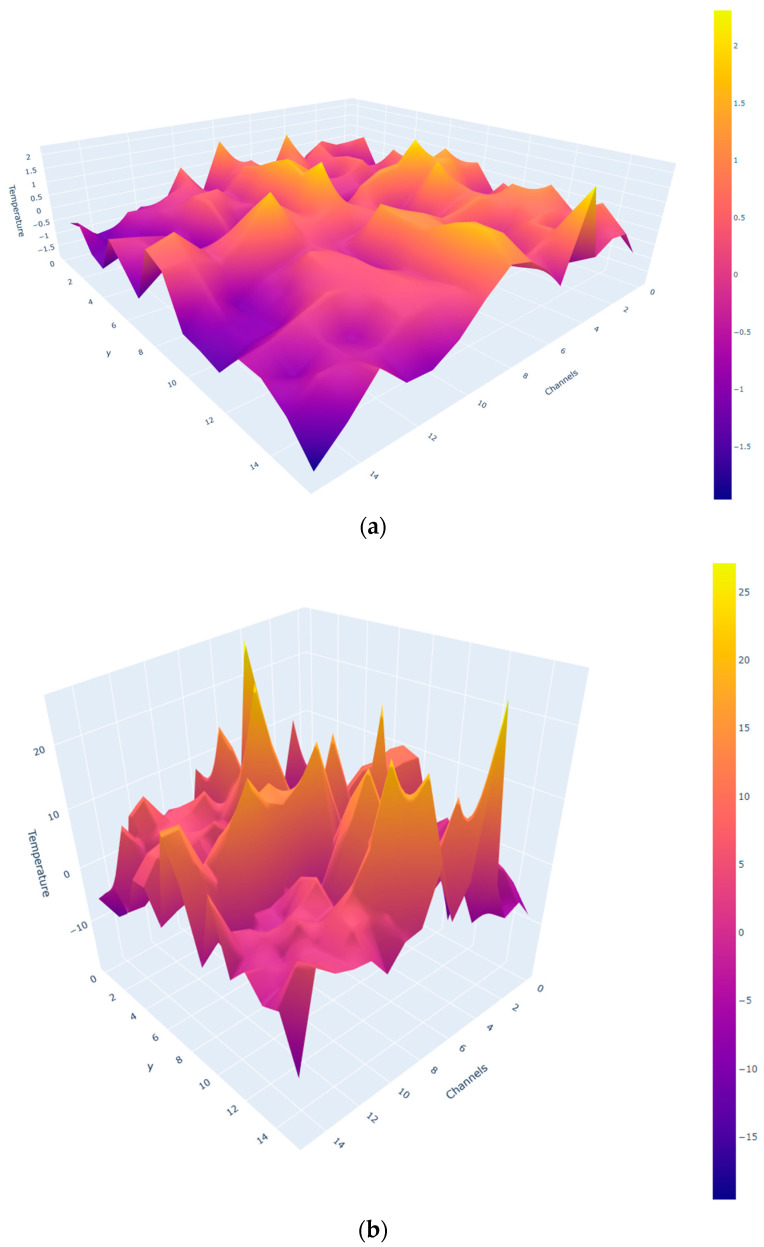
(New cavity—old cavity) 3D heating-difference results (**a**) at 30 s and (**b**) at 360 s.

These experiments documented the temperature changes over time, providing a detailed temporal analysis of the heating process. Figure 10 and Figure 11 show 2D and 3D representations of the differences between the new cavity minus the old cavity’s temperatures, respectively. Two extreme cases of 30 s and 360 s are represented in these Figures.

The old cavity exhibited a plus-shaped pattern of uneven heating in the center due to its ventilation design. The new cavity, with an improved ventilation system, resulted in more homogeneous heating. The temperature differences between the new and old cavities highlights these changes.

### 3.3. Fire Detector Measurements

The first step of the measurements has been dedicated to advancing the development of fire detectors that utilize Bragg gratings coated with a black body, optimized for efficient infrared radiation detection. The primary focus was enhancing the fabrication process of functional coatings. Concerns were raised about the robustness of the deposition process around the sensor location (the layer did not adhere well to the silica surface). To address this issue, the fabrication process was optimized using a sol–gel technique, providing a reproducible deposition of the coating with controlled thickness.

The next steps were refining the housing design for the coated sensors and executing functional tests to assess detector performance. A novel housing design was developed to shield the sensor from dust and optimize the capture of infrared radiation. This housing utilizes a half-pipe aluminum tube as a concentrator combined with capillary tubing, which exploits the greenhouse effect to enhance sensor response. Packaged fire detectors underwent rigorous testing in line with ISO norm 8421 using controlled ethanol or heptane fires. These tests aimed to validate the response time and sensitivity of the sensors, focusing on their performance in both bare and packaged configurations. In the design and implementation of fire detection systems within industrial settings, the positioning of the sensor casing is crucial for ensuring comprehensive coverage and maximum sensitivity. In this project, the casing for the fire sensor was deliberately placed at the middle top of the cavity. This strategic placement was chosen to capitalize on several key factors that contribute to the effective monitoring of fire conditions throughout the oven cavity.

**Figure 12:**
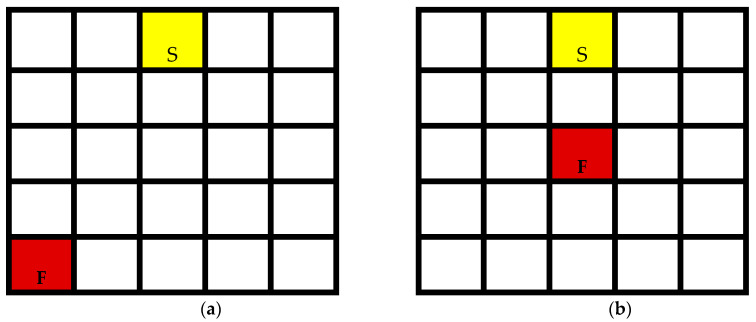
Fire sensor (S) and test fire location (F). (The oven cavity is divided into 5 × 5 grids of equal surface areas). (**a**) Fire at maximum distance; (**b**) aligned fire test.

Initial tests of the infrared sensor in an industrial environment were carried out inside the microwave oven, using small flames such as those from a lighter. They revealed that the detection time was just over 10 s, especially when the flame was far from the detector (see Figure 12a). Since radiation power is a function of the inverse square of distance, the sensor receives only a small amount of infrared radiation, which slows down detection. For smaller distances or larger flames, detection time decrease up to less than 2 s (cf. Figure 12b). These very promising results show that there is still room for improvement, for example, in the radiation concentrator behind the fiber sensor or in the use of materials that are more transparent to infrared radiation to protect the sensor.

While improvements were noted with the use of an IR lamp for controlled testing, the real-world application of fire detectors revealed limitations in detection speed, particularly with smaller flames at extended distances. These findings underscore the need for ongoing optimization of sensor sensitivity and placement within the system to meet all operational safety requirements. Future directions that could be explored: implementation of a shorter uncoated FBG and a longer coated FBG. For the current project, both FBGs have the same length (3–4 mm).

## 4. Conclusions

The “fiber-optic-sensor-assisted safe industrial microwave heating system” has made substantial strides in enhancing the safety and efficiency of industrial microwave heating systems through the integration of advanced sensor technologies. The methodologies employed in this work ensure a comprehensive evaluation of the integrated fiber-optic sensors and fire detection system.

The deployment of fiber Bragg gratings (FBGs) for 2D temperature mapping has provided unprecedented visibility into the thermal profiles within microwave cavities. The detailed temperature maps generated by these sensors allowed for the identification and mitigation of hot spots, which are critical for improving product quality and uniformity in heating processes. The comparison of old and new cavity designs highlighted improvements in energy efficiency and heat distribution, underscoring the effectiveness of the project’s engineering solutions.

For fire detection, most methods in the industry are based on detecting either smoke or heat. Due to the electromagnetic and electric fields inside the cavity, most sensors cannot operate reliably in these conditions. The detection time for our method was proven to be adequate for microwave ovens, where an alternative heat-based sensor would take several seconds or even minutes to trigger an alarm after everything heats up to a certain threshold. The optical fiber fire detectors, coated with materials sensitive to infrared radiation, improved the system’s ability to rapidly detect flames and excessive heat. The development of these sensors not only enhanced the system’s safety by enabling quicker shutdown procedures but also reduced the risk of fire-related incidents, which are significant concerns in high-temperature environments.

The integration of these sensor technologies holds the potential for widespread adoption across various sectors that rely on microwave heating [19,20]. Industries such as food processing, materials manufacturing, and chemical synthesis could benefit significantly from the enhanced monitoring and safety features developed through this project. Looking forward, this project’s approach to integrating real-time monitoring systems opens new possibilities for the development of smart industrial appliances. These systems could potentially adjust operational parameters dynamically based on sensor inputs, leading to greater efficiencies and adaptive safety mechanisms.

## Data Availability

Data are contained within the article.
